# Association between five types of Tumor Necrosis Factor-α gene polymorphism and hepatocellular carcinoma risk: a meta-analysis

**DOI:** 10.1186/s12885-020-07606-6

**Published:** 2020-11-23

**Authors:** Citrawati Dyah Kencono Wungu, Fis Citra Ariyanto, Gwenny Ichsan Prabowo, Soetjipto, Retno Handajani

**Affiliations:** 1 Department of Medical Biochemistry, Faculty of Medicine, Universitas Airlangga, Surabaya, Indonesia; 2 Institute of Tropical Disease, Universitas Airlangga, Surabaya, Indonesia; 3 Faculty of Nursing, Universitas Jember, Jember, Indonesia; 4 Hearing Vision Ltd-Darmo General Hospital, Surabaya, Indonesia

**Keywords:** Meta-analysis, Tumor necrosis factor-α, Single nucleotide polymorphism, Hepatocellular carcinoma

## Abstract

**Background:**

Research focusing on the relationship between five types of tumor necrosis factor-alpha (TNF-α) SNPs and the risk of hepatocellular carcinoma (HCC) were still controversial. Hereby, we performed a meta-analysis to determine the association between TNF-α promoter SNPs: -1031 T/C, − 863 C/A, − 857 C/T, − 308 G/A, and − 238 G/A with HCC risk.

**Methods:**

We interrogated articles from journal database: PubMed, Pro-Quest, EBSCO, Science Direct, and Springer to determine the relationship between five types of SNPs in TNF-α gene with HCC risk. RevMan 5.3 software was used for analysis in fixed/random effect models.

**Results:**

This meta-analysis included 23 potential articles from 2004 to 2018 with 3237 HCC cases and 4843 controls. We found that SNP − 863 C/A were associated with a significantly increased HCC risk (A vs C, OR = 1.31, 95% CI = 1.03–1.67). Similar results were obtained in − 857 C/T (TT/CT vs CC, OR = 1.31, 95% CI = 1.06–1.62), − 308 G/A (AA vs GG, OR = 3.14, 95% CI = 2.06–4.79), and − 238 G/A (AA vs GG, OR = 3.87, 95% CI = 1.32–11.34). While no associations were observed between SNP TNF-α − 1031 T/C and HCC risk.

**Conclusions:**

The present meta-analysis showed that TNFα SNPs -863C/A, − 857 C/T, − 308 G/A, and − 238 G/A were associated with the risk of HCC.

**Supplementary Information:**

The online version contains supplementary material available at 10.1186/s12885-020-07606-6.

## Introduction

Hepatocellular carcinoma (HCC) or liver cancer accounts for the cancer with the fourth highest mortality rate in the world. In 2018, there are estimated to be 841,080 of HCC new cases worldwide [1]. Patients with HCC usually have poor prognosis and high mortality rates, even in developed countries [2]. Until now, the pathogenesis of HCC has not been fully understood, but it is known so far that it is influenced by hepatitis B and C virus infections and also influenced by environmental factors (smoking, alcohol, aflatoxin B1) [3]. It is known that there are differences in the risk of HCC within each person, in which case the host factor has an important role [4].

Tumor Necrosis Factor-α (TNF-α) is an important inflammatory cytokine in the development of liver disease. This cytokine can cause hepatic injury, cirrhosis and eventually promote hepatocellular carcinoma [5, 6]. Several previous studies have identified some Single Nucleotide Polymorphism (SNP) s in TNF-α gene, especially in the promoter region. SNPs of TNF-α -1031 T/C (rs1799964), −863C/A (rs1800630), −857C/T (rs1799724), −308G/A (rs1800629), and -238G/A (rs361525) are SNPs in the TNF-α promoter site that have often been investigated regarding their association with HCC in several previous studies [7, 8]. It was also said that those SNPs could affect TNF-α production at the transcription level [9, 10]. This plays important role because cytotoxic T lymphocytes (CTLs) in the liver secrete TNF-α [11]. Increased TNF-α level due to those SNPs can cause persistent inflammatory condition in liver tissue which is the most important risk factor for HCC [7].

High production of TNF-is related to the increase of pro-inflammatory cytokine secretion, the activation of proto oncogenes and several genes associated with cell growth, invasion, and cancer cells metastasis [12, 13]. Excessive production of TNFα can also induce the generation of free radicals in the form of Reactive Oxygen Species which can cause further liver damage and genomic instability [14]. It is also said that high TNF-α expression is an independent predictor of poor survival in HCC patients [15].

The results of various previous studies regarding the relationship between TNF-α polymorphism and HCC across various ethnicities and populations show mixed results, related [16–18] or there is no relationship [19, 20]. Research on TNF-α gene SNPs in patients with Hepatitis B Virus (HBV) and Hepatitis C Virus (HCV) infection is specific for each ethnicity and shows different results in each population [21, 22]. As the results regarding the role of these five SNPs of TNF-α genes against HCC are still controversial, we conducted this meta-analysis to determine the relationship between those TNF-α gene polymorphisms and HCC.

## Methods

### Database searching

Our meta-analysis was reported based on the items outlined in the Preferred Reporting Items for Systematic Reviews and Meta-Analyses (PRISMA) statement to ensure adequate reporting of this meta-analysis of observational studies in epidemiology [23]. We conducted an electronic database searching to identify all previously published cohort or case-control studies investigating the association between five types of TNF-α gene SNPs (− 1031 T/C, −863C/A, −857C/T, −308G/A, and -238G/A) and risk of HCC. We searched data from PubMed, ProQuest, EBSCO, Science Direct, and Springer by using MeSH terms: “Tumor Necrosis Factor-alpha” or “TNF-alpha” and “polymorphism” or “SNP” or “single nucleotide polymorphism” or “variant” and “hepatocellular carcinoma” or “HCC” or “liver cancer”. The search was conducted in September 2019–January 2020. We also performed a manual search was also performed to obtain potential sources cited in other meta-analysis.

### Criteria for inclusion and exclusion

All included studies should meet the following criteria: (1) investigate the association between any of the five SNPs (− 1031 T/C, −863C/A, −857C/T, −308G/A, and -238G/A) in TNF-α gene and risk of HCC; (2) have cohort or case-control study design; (3) risk of HCC was reported as RR (relative risk) and/or OR (odds ratio) with 95% Confidence Interval (CI) or provide sufficient data to extract RR and/or OR with 95% CI data; (4) include human subjects; (6) in English language. We excluded studies with the following criteria: (1) studies with design other than case-control or cohort; (2) duplicated studies; (3) studies with unmeasurable population and not qualified data; (4) using non-English language; (5) Studies in which the full text or main data could not be obtained.

### Study selection

Two investigators independently performed the electronic search and retrieved the articles that matched with our searched terms. Any disagreement was settled by discussion and consensus with all the authors. Final decision was merely based on the agreements of all authors.

### Data extraction

A standardized reporting form was used to extract the data from each article which included the first author’s name, year of publication, population country, TNF-α SNP type, study design, etiology of HCC, SNP genotyping method, controls, and frequencies of SNP. Hardy–Weinberg equilibrium test was performed and its significance of the control groups was calculated when the original information was not provided.

### Quality assessment

Newcastle - Ottawa quality assessment scale (NOQS) was used for measuring the quality of the included studies. This scale was designed through collaboration between the Universities of Newcastle, Australia and Ottawa, Canada. The purpose of this scale was to assess the quality of observational studies for producing good meta-analysis. The studies were qualified as high quality (9 stars), medium quality (7–8 stars), and low quality (less than 7 stars) [24].

### Data analysis

Analysis were conducted using Review Manager 5.3 software (The Cochrane Collaboration, UK). Hardy-Weinberg Equilibrium was examined by Chi Square test when the original information was not provided. We evaluate 5 genetic models (allele, dominant, recessive, codominant major vs minor homozygote, and codominant heterozygote vs major homozygote) for each SNP type separately. Major alleles for each SNP were: -1031TT, −863CC, −857CC, −308GG, and -238GG, while minor alleles for each SNP were: -1031CC, −863AA, −857TT, −308AA, and -238AA. Heterogeneity assumption was assessed with Cochrane Q statistic and I_2_ statistic. The pool estimated ORs was calculated with either fixed or random effects model assumptions. If Q test showed significant result (*p* < 0.05), we used a random effects model. Otherwise, if Q test showed insignificant result (*p* > 0.05), we used a fixed effect model. We also calculated the 95% confidence interval (CI) of pool estimated OR. Inverted funnel plots were conducted to find any presence of publication bias.

## Results

### Study selection

**Fig. 1:**
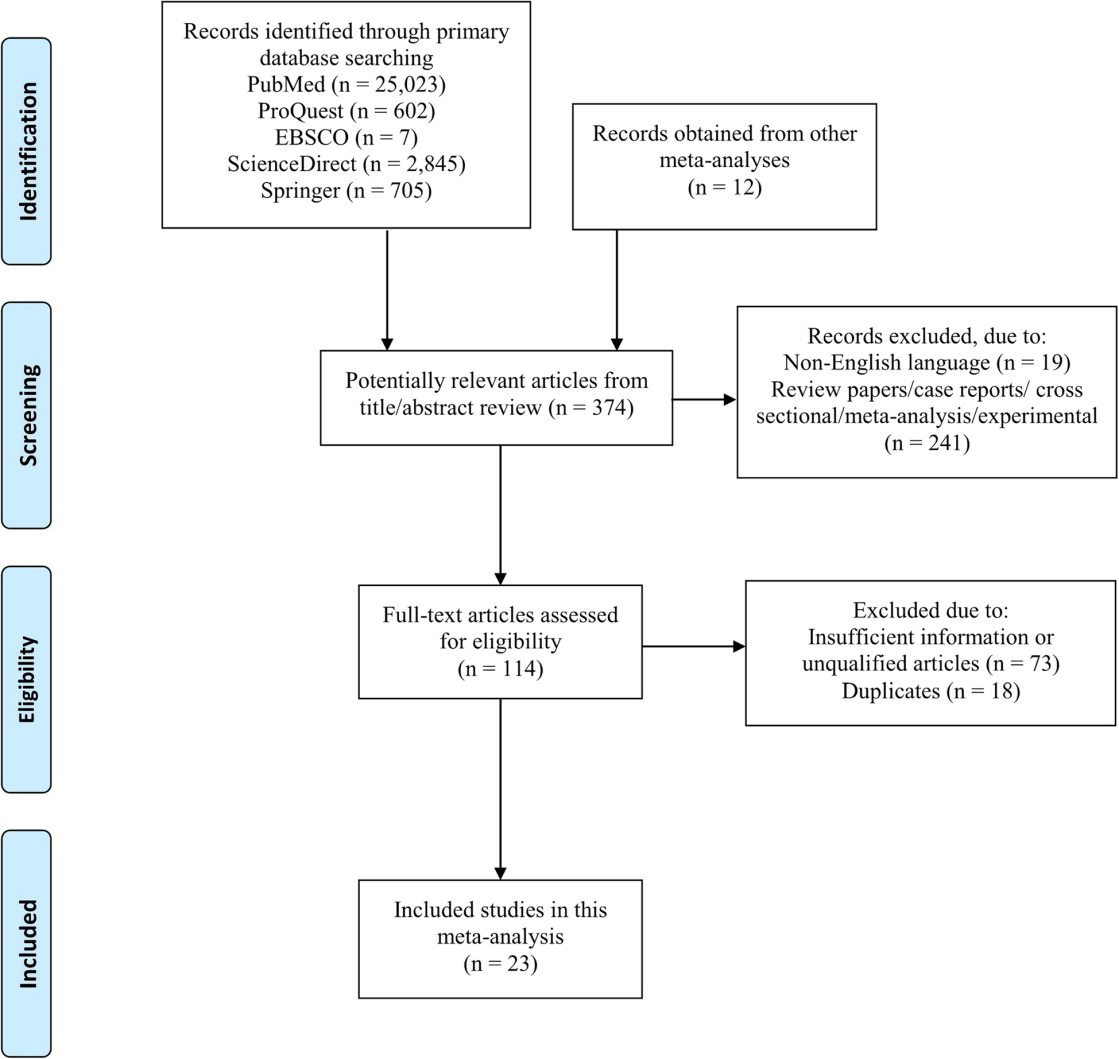
PRISMA diagram of the literature search

According to the PRISMA flow diagram (Fig. 1), we initially obtained 29,182 studies through primary database searching and 12 through manual searching. After screening the titles/abstracts we selected 374 potentially relevant articles. Among them, 260 were excluded due to non-English language (19 studies) and unsuitable study designs (review papers/case reports/ cross sectional/meta-analysis/experimental; 241 studies). Then, 114 full text studies were checked for their eligibility. Some studies were excluded due to duplicates or irrelevant study design/insufficient information/unqualified articles until we finally obtained 23 included studies.

Eventually, as much as 23 potential articles were included which recruited 9792 participants and consisted of 3237 HCC cases and 4843 controls (Table 1). The papers were published 2004 to 2018. Each study has sample size ranged from 45 to 1624 participants. Of all included studies in this meta-analysis, most studies came from Asia, in which 7 studies were from Mainland China [7, 25–30], 4 from Taiwan [16, 31–33], 2 from India [19, 34], 2 from Korea [8, 35], 1 from Japan [36], 1 from Thai [37], and 1 from Turkey [38]. There were also some studies with non-Asian countries, including 2 from Egypt [39, 40], 1 from Brazil [41], 1 from Italy [42], and 1 from Tunisia [43]. From the 23 included studies, the aetiology of HCC was mostly caused by HBV (12 studies), followed by mixed cause (8 studies), HCV (2 studies), and alcohol/smoking (1 study). Some studies observed 1 locus, and others observed more than 1 loci. Studies observing polymorphism at − 308 was the most frequent (19 studies), while SNP − 1031 was the less frequently observed (5 studies). Allele frequencies of SNPs in each population is showed on Table 2.

**Table 1:** The basic characteristics of the included studies

No	First author	Year	TNF-α SNPs	Study design	Sample size		Population	SNP genotyping method	HCC etiology	Control	NOQS
					Case (*n* = 3237)	Control (*n* = 4843)					
1	Heneghan	2004	-308 G/A, −238 G/A	Case control	98	75	China	PCR-RFLP	Mixed	Healthy subjects	8
2	Ho	2004	-308 G/A	Case control	74	289	Taiwan	PCR-RFLP	Mixed	Healthy subjects	8
3	Migita	2005	-308 G/A	Case control	48	188	Japan	PCR-SSP	HBV	HBV without HCC	7
4	Niro	2005	−1031 T/C, −863 C/A, −308 G/A, −238 G/A	Case control	30	96	Italy	Sequencing	HBV	SR	7
5	Jeng	2007	−308 G/A	Case control	108	108	Taiwan	Sequencing	Mixed	Healthy subjects	9
6	Kummee	2007	−863 C/A, −308 G/A, −238 G/A	Case control	50	150	Thailand	PCR-RFLP	HBV	Healthy subjects	7
7	Akkiz	2009	−308 G/A	Case control	110	110	Turkey	PCR-RFLP	Mixed	Healthy subjects	9
8	Wang	2010	−308 G/A, −238 G/A	Case control	230	158	China	Sequencing	HBV	SR	8
9	Chen	2011	−1031 T/C, −863 C/A, −857 C/T, −238 G/A	Case control	126	126	China	Sequencing	HBV	Healthy subjects	9
10	Shi	2011	−308 G/A	Case control	88	88	China	PCR-RFLP	HBV	Healthy subjects	9
11	Qiu	2012	−863 C/A, −857 C/T	Case control	195	189	China	PCR-RFLP	HBV	SR	7
12	Radwan	2012	−308 G/A	Case control	128	160	Egypt	PCR-RFLP	HCV	Healthy subjects	8
13	Shi	2012	−308 G/A	Case control	73	116	China	PCR-RFLP	Mixed	Healthy subjects	8
14	Yang	2012	−863 C/A	Case control	772	852	China	RT-PCR	Mixed	Healthy subjects	7
15	Teixeira	2013	−308 G/A, −238 G/A	Case control	111	202	Brazil	PCR-SSP	Mixed	Healthy subjects	5
16	Panigrahi	2014	−863 C/A, −857 C/T, −238 G/A	Case control	14	85	India	PCR-RFLP	HBV	Healthy subjects	6
17	Saxena	2014	−308 G/A	Case control	59	139	India	PCR-RFLP	HBV	Healthy subjects	8
18	Jin	2015	−1031 T/C, −857 C/T, −238 G/A	Case control	224	206	Korea	Single base primer extension assay	HBV	LC	7
19	Sghaier	2015	−308 G/A, −238 G/A	Case control	15	200	Tunisia	PCR-RFLP	HBV	Healthy subjects	8
20	Shin	2015	−1031 T/C, −863 C/A, −857 C/T, −308 G/A, −238 G/A	Case control	157	201	Korea	PCR-RFLP	Mixed	Healthy subjects	8
21	Yang	2015	−1031 T/C, −863 C/A, −857 C/T, −308 G/A	Case control	298	889	Taiwan	PCR-RFLP	Smoking and alcohol	Healthy subjects	7
22	Tsai	2017	−308 G/A	Case control	200	200	Taiwan	Sequencing	HBV	LC	6
23	Tharwat	2018	−308 G/A	Retrospective cohort	29	16	Egypt	PCR-RFLP	HCV	Healthy subjecys	8

*ASC* Asymptomatic Carrier, *LC* Liver Cirrhosis, *HCC* Hepatocellular Carcinoma, *PCR-RFLP* Polymerase chain reaction-restriction fragment length polymorphism, *PCR-SSP* Polymerase chain reaction-sequence-specific primers, *RT-PCR* Real-time polymerase chain reaction, *NOQS* Newcastle-Ottawa quality assessment scale

**Table 2:** Allele frequencies of SNPs in each population

SNP	Eastern Asia		Southern Asia		Africa		Europe		South America	
	HCC	Control	HCC	Control	HCC	Control	HCC	Control	HCC	Control
-1031C	22.12%	21.75%	–	–	–	–	–	–	–	–
-863A	15.67%	14.62%	39%	32.98%	–	–	–	–	–	–
-857 T	19.25%	18.21%	14.29%	12.94%	–	–	–	–	–	–
-308A	13.68%	8.73%	6.88%	6.75%	25.58%	26.33%	18.64%	5%	13.51%	7.92%
-238A	5.97%	4.64%	9.38%	8.51%	66.67%	45.75%	–	–	18.47%	9.65%

### Association between SNP TNF-α − 1031 and HCC risk

Only five studies regarding the association between SNP TNF-α − 1031 and HCC risk with 825 cases and 1518 controls were available. The number of cases for CC and TC genotypes was reported together in the studies by Niro et al. [42] and Jin et al. [35] which could only be used for dominant-model analysis (CC/TC vs TT; Table 3). As the heterogeneity among studies for all models (I_2_) was less than 60% and *p*-value for the heterogeneity was more than 0.05, fixed-effects models were applied. However, we did not obtain any significant association between SNP TNF-α − 1031 and HCC risk in all model analyses. For the estimation of publication bias, we found no visual asymmetry in Funnel Plot analysis.

**Table 3:** Pooled risk estimates for SNPs TNF-α −1031 T/C, −863 C/A, −857 C/T, −308 G/A, and − 238 G/A with HCC risk

SNP TNF-α	n	OR (95% CI)	*p* value for Z test	I^**2**^ for heterogenity	*p* value for heterogenity
**−1031 T/C**					
C vs T	3	1.06 [0.89–1.26]	0.52	0.77	0
CC + TC vs TT	5	1.04 [0.86–1.27]	0.69	0.97	0
CC vs TT	3	1.39 [0.88–2.18]	0.15	0.99	0
CC vs TC + TT	3	1.41 [0.91–2.21]	0.13	1	0
TC vs TT	3	0.96 [0.77–1.19]	0.70	0.64	0
**−863 C/A**					
A vs C	7	1.31 [1.03–1.67]	0.03*	0.007	66
AA+CA vs CC	8	1.19 [1.03–1.36]	0.02*	0.35	10
AA vs CC	7	1.43 [0.98–2.10]	0.07	0.50	0
AA vs CA + CC	7	1.28 [0.89–1.86]	0.19	0.84	0
CA vs CC	**7**	1.41 [1.00–1.99]	0.05	0.0007	74
**−857 C/T**					
T vs C	4	1.16 [0.94–1.43]	0.17	0.45	0
TT + CT vs CC	5	1.31 [1.06–1.62]	0.01*	0.24	27
TT vs CC	4	0.75 [0.42–1.36]	0.35	0.87	0
TT vs CT + CC	4	0.77 [0.44–1.35]	0.36	0.77	0
CT vs CC	4	1.02 [0.77–1.34]	0.89	0.54	0
**−308 G/A**					
A vs G	17	1.98 [1.62–2.42]	< 0.001*	0.03	44
AA+GA vs GG	19	1.95 [1.53–2.49]	< 0.001*	0.003	54
AA vs GG	13	3.14 [2.06–4.79]	< 0.001*	0.69	0
AA vs GA + GG	13	2.52 [1.69–3.76]	< 0.001*	0.81	0
GA vs GG	17	2.07 [1.60–2.68]	< 0.001*	0.006	52
−**238 G/A**					
A vs G	8	1.50 [1.16–1.94]	0.002*	0.07	46
AA+GA vs GG	9	1.39 [0.87–2.24]	0.17	0.03	52
AA vs GG	5	3.87 [1.32–11.34]	0.01*	0.70	0
AA vs GA + GG	8	2.67 [1.17–6.10]	0.02*	0.87	0
GA vs GG	8	1.28 [0.72–2.28]	0.39	0.01	61

Note: *Significant *p* < 0.05

### Association between SNP TNF-α − 863 and HCC risk

**Fig. 2:**
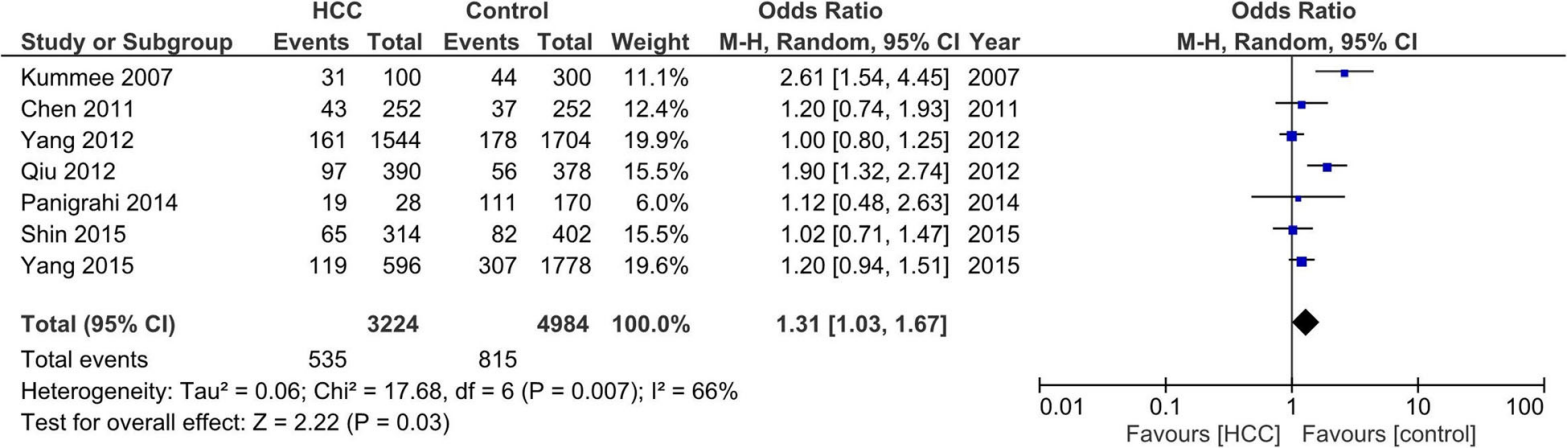
Allele model (A vs C) of SNP TNF-α 863C/A on HCC risk. The association was indicated as odds ratio (OR) estimate with 95% CI. Odd Ratio > 1 shows the increased risk of HCC

We included eight studies with 1642 cases and 2746 controls to determine the association between SNP TNF-α − 863 and HCC risk. The numbers of cases for CA and AA genotypes were reported together in the study by Niro et al. [42], thus it could only be used for dominant-model analysis (AA/CA vs CC; Table 3). In studies with heterogeneity among studies (I_2_) more than 60% and *p*-value for the heterogeneity was less than 0.05, we applied random-effects models. We found significant association between the allele model of A versus C of TNF-α C/A SNP with the risk of HCC (OR = 1.31, 95% CI = 1.03–1.67, *p* = 0.03). The dominant-model analysis (CA/AA vs CC) also showed significant association between SNP TNF-α 863 C/A and HCC risk (OR = 1.19, 95% CI = 1.03–1.36, *p* = 0.02; Fig. 2). As heterogeneity was found in the statistical analyses, we did sensitivity analyses to evaluate the sources of heterogeneity. We found that heterogeneity between studies was mainly caused by the study by Kummee et al. [37], as after this study was excluded, no significant heterogeneity was found.

### Association between SNP TNF-α − 857 and HCC risk

**Fig. 3:**
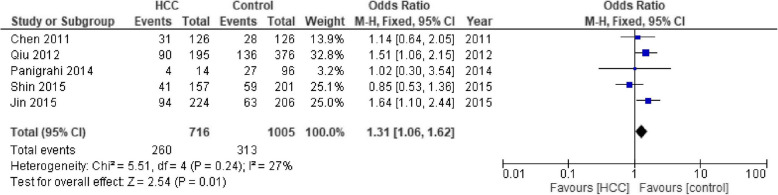
Dominant-model analysis (TT/CT vs CC) of SNP TNF-α 857 C/T on HCC risk

We only obtained five studies about the association between SNP TNF-α − 857 and HCC risk with 716 cases and 1005 controls. The number of cases for TT and CT genotypes was reported together in the studies by Jin et al. [35] which could only be used for dominant-model analysis (TT/CT vs CC; Table 3). As the heterogeneity among studies for all models (I_2_) was less than 60% and *p*-value for the heterogeneity was more than 0.05, fixed-effects models were applied. A significant association between SNP TNF-α − 857 C/T and HCC risk was found in dominant-model analyses (OR = 1.31, 95% CI = 1.06–1.62, *p* = 0.01; Fig. 3). We did not observe any visual asymmetry in Funnel Plot analysis regarding the publication bias.

### Association between SNP TNF-α − 308 and HCC risk

**Fig. 4:**
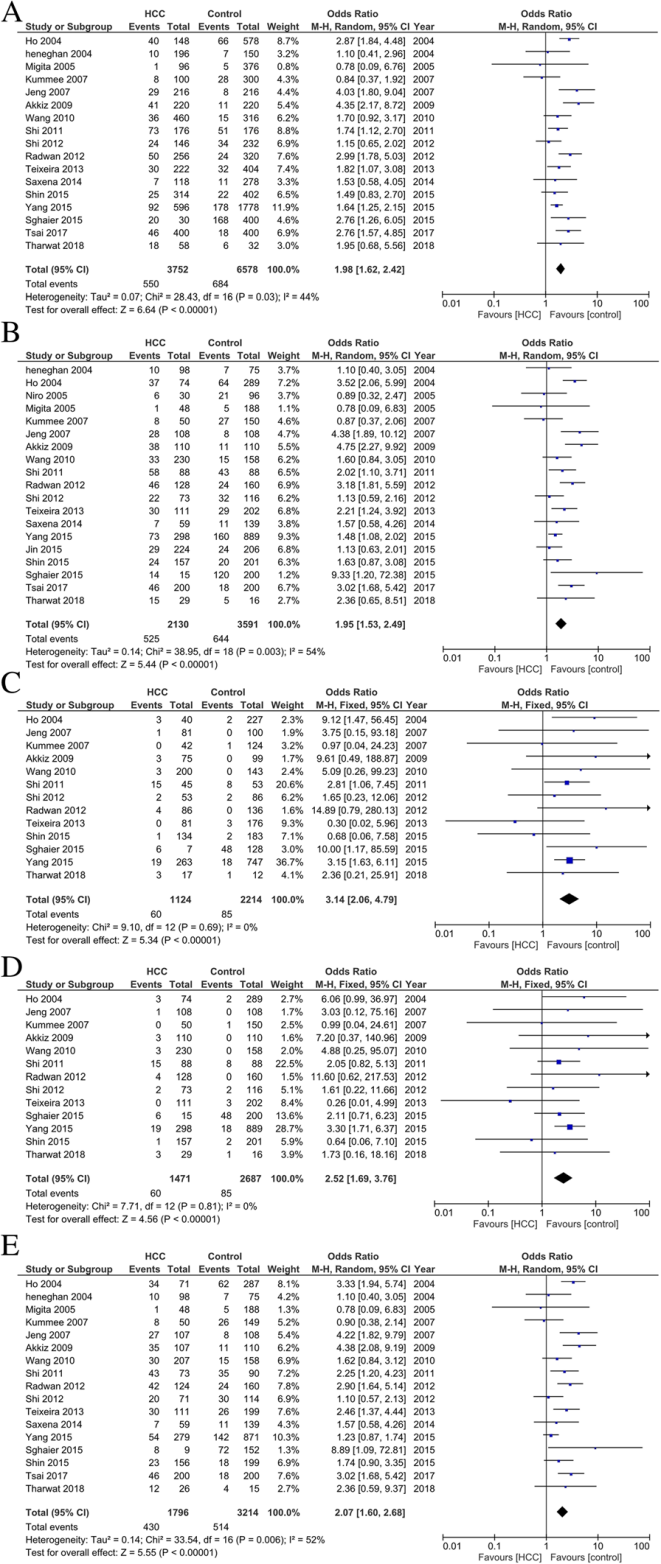
(a) Allele model analysis (A vs G), (b) Dominant model analysis (AA+GA vs GG), (c) Codominant major vs minor homozygote-model analysis (AA vs GG), (d) Recessive-model analysis (AA vs GA+GG), and (e) codominant heterozygote vs major homozygote model analysis (GA vs GG) of SNP TNF-α 238 G/A on HCC risk.

Interestingly, we found most included studies analyzing the association between SNP TNF-α − 308 G/A and HCC risk. The number of cases for AA and GA genotypes was reported together in the studies by Niro et al. [42] and Jin et al. [35] which could only be used for dominant-model analysis (AA/GA vs GG; Table 3). In studies with heterogeneity among studies (I_2_) more than 60% and *p*-value for the heterogeneity was less than 0.05, random-effects models were applied. All five genetic models showed significant association with risk of HCC with OR for allele model = 1.98, 95% CI = 1.62–2.42, *p* < 0.001; OR for dominant model = 1.95, 95% CI = 1.53–2.49, *p* < 0.001; OR for recessive model = 2.52, 95% CI = 1.69–3.76, *p* < 0.001; OR for codominant major vs minor homozygote model = 3.14, 95% CI = 2.06–4.79, *p* < 0.001; and OR for codominant heterozygote vs major homozygote model = 2.07, 95% CI = 1.60–2.68, *p* < 0.001; Fig. 4). As heterogeneity was found in the statistical analyses, we did sensitivity analyses to evaluate the sources of heterogeneity. We found that heterogeneity between studies was mainly caused by the studies by Ho et al. [31] and Akkiz et al. [38], as after these studies were excluded, no significant heterogeneity was found.

### Association between SNP TNF-α − 238 and HCC risk

**Fig. 5:**
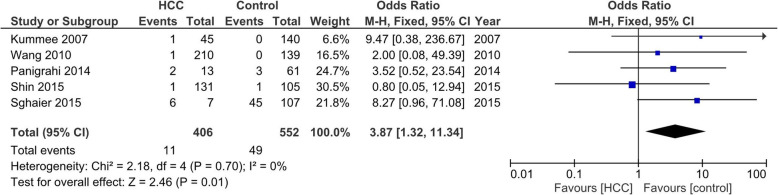
Codominant-model analysis (AA vs GG) of SNP TNF-α 238 G/A on HCC risk

Nine studies with 831 cases and 1293 controls were included to determine the association between SNP TNF-α − 238 and HCC risk. Similar to the TNF-a − 863 SNP, the numbers of cases for GA and AA genotypes were reported together in the study by Niro et al. [42], thus it could only be used for dominant-model analysis (GA/AA vs GG; Table 3). In studies with heterogeneity among studies (I_2_) more than 60% and *p*-value for the heterogeneity was less than 0.05, we applied random-effects models. The allele model of A versus G of SNP TNF-α − 238 G/A was significantly associated with risk of HCC (OR = 1.50, 95% CI = 1.16–1.94, *p* = 0.002). The codominant-model analysis (AA vs GG) also showed significant association between SNP TNF-α 238 G/A and HCC risk (OR = 3.87, 95% CI = 1.32–11.34, *p* = 0.01). The recessive model analysis (AA vs GA + GG) proved significant association between SNP TNF-α 238 G/A and HCC risk as well (OR = 2.67, 95% CI = 1.17–6.10, *p* = 0.02; Fig. 5). As heterogeneity was found in the statistical analyses, we did sensitivity analyses to evaluate the sources of heterogeneity. We found that heterogeneity between studies was mainly caused by the study by Teixeira et al. [41], as after this study was excluded, no significant heterogeneity was found.

### Sensitivity analysis

In this meta-analysis, there were no significant change in ORs by deleting a particular study, which indicated that no single study influenced the statistical significance of the overall results.

## Discussion

The development of HCC depends on some factors such as viral infection, environmental, behavioral (smoking, alcoholism), metabolism, and genetics [44, 45]. The contribution of SNP as a genetic factor is widely studied related to its role in the development of HCC, in which the prevalence of SNP is different in each population [5, 46, 47]. From the host factor, TNF-α is thought to play an important role in hepatocarcinogenesis through necroinflammation and the induction of fibrogenic factors [48]. Five biallelic SNPs in the promoter region Tumor Necrosis Factor-α gene were known: -1031 T/C, −863C/A, −857C/T, −308G/A, and -238G/A [7]. However, to date, only few meta-analyses have analysed all these five SNPs with HCC risk. Hereby, we performed this updated meta-analysis to clarify the independent role of each of these five SNPs on HCC risk.

As research on SNP TNF-α − 1031 T/C and HCC risk is still limited, we only found five studies investigating this SNP, even though many studies have shown significant association between this SNP with other diseases such as polycystic ovary syndrome and endometriosis [49, 50]. In a study conducted by Shin et al. survival of HCC cases with TNF-α − 1031 wild type (TT) genotype or SNP TC genotype was significantly better than those with the SNP CC genotype [8]. However, in this meta-analysis, no significant relationship was found between the SNP and HCC risk in all genetic models. Meta-analysis conducted by Wei et al. also shows that there is no significant relationship between this SNP and HCC risk [51].

This study shows a significant relationship between SNP TNF-α − 863 C/A with HCC risk in allele models and dominant model analysis. A meta-analysis conducted by Wei et al. also showed a significant relationship betweehis SNP and HCC in dominant and codominant (CA vs CC) model analysis [51]. Polymorphism of TNF-α − 863 C/A in the promoter region can influence TNF-α expression, however, the result is still conflicting. Some research suggest that it may increase TNF-α expression [52, 53], while other research show the opposite result in which it may decrease TNF-α expression [30, 54]. A study by Skoog et al. proposed that TNF-α − 863 C/A polymorphism affects the binding of nuclear protein(s) to the promoter region of the TNF-α gene, with accompanying changes in TNF-α expression, thus leading to variation in TNF-α levels [55]. The role of ethnicity may also play a role as well, carriers of the rare ‘A’ allele have a significantly lower TNF-α levels in Swedish and Indian population [55, 56], while they have a significantly higher levels in Japanese population [52, 53].

In the present study, we also found a significant relationship between SNP TNF-α − 857 C/T with dominant model analysis. Limited studies were present regarding the relationship between this polymorphism with HCC risk, thus there were only 7 studies that came within our inclusion criteria. This is different from the meta-analysis conducted by Wei et al. which showed no significant relationship between this SNP and HCC risk, however in that meta-analysis there were only 3 included studies [51].

There have been many previous studies investigating SNP TNF-α − 308 G/A with HCC risk, thus we got 19 included studies. All five genetic analysis models showed a significant relationship between the SNP and HCC risk. This is in line with several previous meta-analysis studies, including those conducted by Hu et al. and Tavakolpour and Sali on allele models and dominant model analyses [17, 57]. Wei et al. on codominant and dominant model analyses [51], and Xiao et al. in all except recessive model analysis [18]. Of all other TNF-α SNPs, − 308 G/A is the most studied SNP. This SNP is also correlated with the risk of other cancer, such as breast cancer and gastric cancer [56, 58]. This SNP seems to be variable within ethnicities, as in meta-analysis study conducted by Wei et al., SNP − 308 AA was associated with an increased risk of HCC in Asian ethnicities, but not for Caucasian [51]. A study conducted by Shin et al. on South Korean population showed that TNF-α − 308 SNP alone was not significantly associated with HCC, but when several genotypes were combined (e.g. -1031 / − 308 / -238 TT / GG / GA), there was a significant association with the incidence of HCC [8].

Studies on SNP TNF-α − 238 G/A have also been extensively performed. Nevertheless, meta-analyses of these SNPs are still limited and yield conflicting results. The present study showed a significant relationship between this SNP and HCC risk. This is in line with a meta-analysis conducted by Xiao et al. showing that this SNP is associated with HCC risk, although in that study only HBV-related HCC was studied [18]. Another meta-analysis conducted by Hu et al., however, shows no significant relationship between this SNP with HCC risk in Asian population [57].

Those SNPs on TNF-α promoter can influence TNF-α level. They resulted in higher and constitutive TNF-α expression and were associated with an increased risk of HCC [10, 17]. TNF-α itself is a potent pro-inflammatory cytokine [17, 59]. Necroinflammation in hepatocytes triggers mutagenesis and activation of oncogenes from proto-oncogenes in host cells, causing HCC [48]. In addition, through the chronic inflammatory pathway, TNF-α is also known to induce HCC through activation and differentiation of hepatic progenitor cells [60].

This meta-analysis still had several limitations. To date, there has rarely been any meta-analysis discussing the relationship between all each SNPs of TNF-α − 1031 T/C, − 863 C/A, − 857 C/T, and − 238 G/A with HCC. The second limitation of this study was that we only included studies in English language so that it does not rule out the exclusion of any good research in non-English languages. Third, the role of other factors, such as variations in genes adjacent to the TNF-α gene or epigenetic factors are said to also be able to regulate TNF-α expression [61, 62]. Fourth, our study is a meta-analysis so it is not possible to generate a per-patient haplotype analysis. Further research is required to determine the effects of these various TNF-α SNPs in each different population.

## Conclusion

In conclusion, this meta-analysis determined the association between SNP TNF-α − 1031 T/C, − 863 C/A, − 857 C/T, − 308 G/A, and − 238 G/A and HCC risk. Our novel data demonstrated that all these SNPs but − 1031 T/C might increase HCC risk. Therefore, genetic predisposition, especially polymorphism of TNF-α gene may play important role in the pathogenesis of HCC. Further studies in larger population and analysing gene-environment interaction are required to provide better understanding between TNF-α polymorphisms and the risk of HCC.

## Supplementary Information

**Additional file 1.**

## Data Availability

The datasets used and/or analyzed during the current study are available from the corresponding author on reasonable request.

## Abbreviations

HCC: Hepatocellular carcinoma
TNF-α: Tumor Necrosis Factor-α
SNP: Single Nucleotide Polymorphism
HBV: Hepatitis B Virus
HCV: Hepatitis C Virus
RR: Relative risk
OR: Odds ratio
CI: Confidence interval
NOQS: Newcastle - Ottawa quality assessment scale
