# Supplementary material for: Association between five types of Tumor Necrosis Factor-α gene polymorphism and hepatocellular carcinoma risk: a meta-analysis
Source: BMC Cancer. 2020 Nov 23;20:1134. doi: 10.1186/s12885-020-07606-6 (PMC7686711; doi:10.1186/s12885-020-07606-6)
Supplement: Supplementary file 1 — Additional file 1. [file 12885_2020_7606_MOESM1_ESM.doc]

Funnel Plot


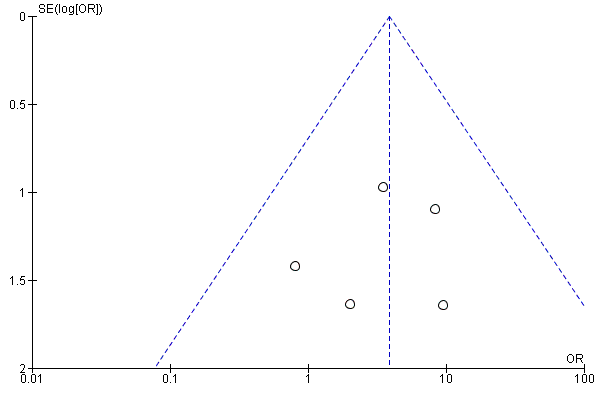


Funnel plot 238 codominant


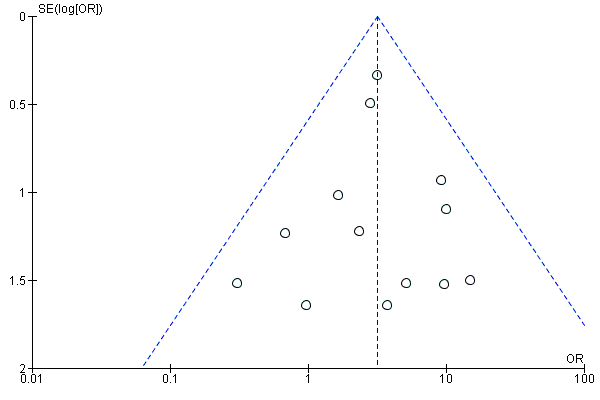


Funnel plot 308 codominant


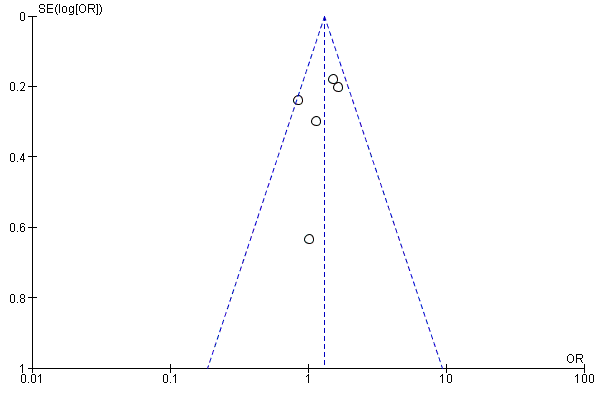
 Funnel plot 857 Dominant


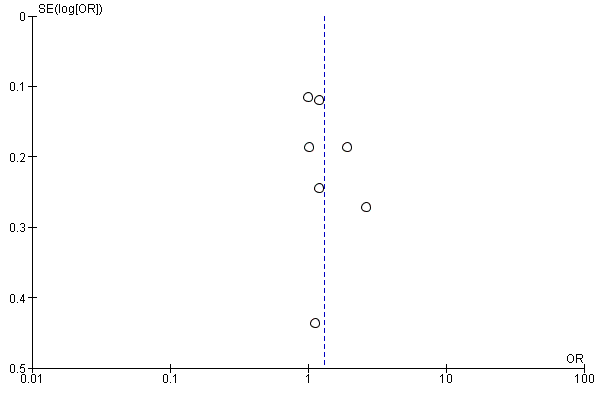


Funnel plot 863 Allele
